# Association between treatment-induced changes in the Kansas City Cardiomyopathy Questionnaire and clinical outcomes in chronic heart failure: a trial-level meta-regression analysis

**DOI:** 10.1016/j.ijcha.2026.101881

**Published:** 2026-01-27

**Authors:** Hidekatsu Fukuta, Toshihiko Goto

**Affiliations:** a Core Laboratory, Nagoya City University Graduate School of Medical Sciences, Nagoya, Japan; b Department of Cardiology, Nagoya City University Graduate School of Medical Sciences, Nagoya, Japan

**Keywords:** Meta-analysis, Meta-regression, Randomized controlled trials, Kansas City Cardiomyopathy Questionnaire, Patient-reported outcome, Regulatory science

## Abstract

**Background:**

Patient-reported outcomes such as the Kansas City Cardiomyopathy Questionnaire (KCCQ) are increasingly recognized for their prognostic value. While the composite endpoint of cardiovascular death and heart failure (HF) hospitalization is the standard primary outcome in contemporary HF trials, the potential for KCCQ changes to serve as an intermediate endpoint for these clinical events requires further evaluation.

**Methods:**

We conducted a trial-level meta-regression analysis of phase 3 randomized controlled trials (RCTs) evaluating pharmacological therapies for chronic HF that reported both changes in KCCQ and the primary composite endpoint of cardiovascular death and HF hospitalization. Weighted random-effects meta-regression models were used to assess the association between changes in KCCQ scores and treatment effects on clinical outcomes.

**Results:**

Twelve phase 3 RCTs were included, comprising eight enrolling patients with HF with reduced ejection fraction and four enrolling those with preserved ejection fraction. Changes in KCCQ scores were significantly associated with treatment effects on the primary composite endpoint (regression coefficient [95% CI] = −0.0611 [−0.0930, −0.0292]; p = 0.001; I^2^ = 2.2%), cardiovascular death (−0.0676 [−0.1099, −0.0254]; p = 0.004; I^2^ = 0%), and HF hospitalization (−0.0700 [−0.1322, −0.0775]; p = 0.031; I^2^ = 54%).

**Conclusion:**

Treatment-induced changes in KCCQ scores are significantly correlated with clinical outcomes in phase 3 HF trials. These findings support KCCQ as a promising candidate intermediate endpoint that provides supportive evidence for the clinical benefit of HF therapies. However, our results remain hypothesis-generating, and further validation using individual patient data and cross-mechanism studies is warranted before KCCQ can be formally established as a regulatory surrogate endpoint.

## 1 Introduction

Heart failure (HF) remains a major cause of morbidity and mortality worldwide, and the efficient development and regulatory approval of novel therapies critically depend on the selection of appropriate clinical endpoints [1]. Hard endpoints such as cardiovascular death and hospitalization for HF are clinically meaningful but require large sample sizes and prolonged follow-up periods [2], [3]. To overcome these challenges, putative surrogate endpoints such as B-type natriuretic peptide (BNP) or N-terminal pro–B-type natriuretic peptide (NT-proBNP), physiological markers of ventricular wall stress and filling pressure, have been widely employed. However, trial-level meta-regression analyses have demonstrated that treatment-induced changes in BNP or NT-proBNP have only limited ability to predict clinical outcomes [4], [5]. Therefore, BNP or NT-proBNP has not been formally recognized by regulatory authorities as a surrogate endpoint for evaluating therapeutic efficacy [6], [7].

In recent years, patient-reported outcomes have gained increasing importance as measures of treatment benefit from the patient’s perspective [3], [8]. Among these, the Kansas City Cardiomyopathy Questionnaire (KCCQ), which captures symptoms, physical function, and quality of life, has been shown to correlate with clinical outcomes [9], [10].

Accordingly, we conducted a trial-level meta-regression of randomized controlled trials (RCTs) of chronic HF that adopted the composite endpoint of cardiovascular death and HF hospitalization as the primary outcome, to examine the association between treatment-induced changes in KCCQ and major clinical outcomes. By assessing this trial-level association, we aimed to evaluate the potential of KCCQ as a candidate intermediate endpoint that could provide supportive evidence for therapeutic efficacy in HF drug development.

## 2 Methods

This meta-analysis was performed according to the Preferred Reporting Items for Systematic Review and Meta-analysis (PRISMA) statement [11]. We systematically identified RCTs involving patients with chronic HF and evaluated the association between changes in KCCQ scores and the primary composite endpoint of cardiovascular death and HF hospitalization.

### 2.1 Study selection

Eligible studies were RCTs that met all of the following criteria: (1) adult patients with chronic HF were enrolled; (2) drug interventions for chronic HF were evaluated; (3) baseline and post-treatment changes in KCCQ scores were reported; and (4) the primary composite endpoint of cardiovascular death and HF hospitalization was reported. Trials of acute HF, pilot studies, phase 2 trials, and non-English language articles were excluded. We restricted our analysis to phase 3 trials with this specific primary composite endpoint to ensure clinical comparability and reduce heterogeneity in treatment effects across the included trials.

### 2.2 Search strategy

Chronic HF trials published until October 31, 2025 were identified using PubMed and Scopus. For search of the eligible studies, the following keywords and Medical Subject Heading were used: *heart failure, Kansas City Cardiomyopathy Questionnaire, and randomized controlled trial.* Literature search was also conducted by manual screening of reference lists of relevant reviews and retrieved articles.

Two researchers (HF and TG) independently performed the literature search. We initially reviewed the titles and abstracts of each study, and if a study was considered relevant, we proceeded to read the full text. Disagreements were resolved by consensus.

### 2.3 Data extraction

From each eligible trial, two reviewers (HF and TG) independently extracted the following information: trial name, year of publication, enrollment ejection fraction (EF), intervention and control treatments, sample size, follow-up duration, hazard ratio (HR) and 95% confidence interval (CI) for the primary composite endpoint and its individual components, the number of patients with available KCCQ data, and the type of KCCQ score with its change from baseline. For trials reporting multiple KCCQ timepoints, we prioritized data between 6 and 12 months (selecting the earliest assessment within this window) and used 4-month data only when no assessments were available between 6 and 12 months. If multiple KCCQ summary scores were reported within the same trial, we prioritized the Clinical Summary Score; when the Clinical Summary Score was not available, the Total Symptom Score or the Overall Summary Score was extracted.

### 2.4 Risk of bias assessment

The risk of bias for each included RCT was assessed using the Cochrane Collaboration’s Risk of Bias tool [12]. Two reviewers (HF and TG) independently evaluated the studies across seven domains: random sequence generation, allocation concealment, blinding of participants and personnel, blinding of outcome assessment, incomplete outcome data, selective reporting, and other biases. For incomplete outcome data, risk was defined as high for primary endpoints if loss-to-follow-up was ≥ 5%. For KCCQ, risk was categorized as low (≥80% attainment), unclear (70–79%), or high (<70%). Discrepancies were resolved by consensus.

### 2.5 Statistical analysis

The associations between treatment-induced changes in KCCQ and treatment effects on the primary composite endpoint and its individual components were assessed at the trial level using weighted random-effects meta-regression analyses conducted on the log HR scale. Each trial was plotted as a single point on a bubble plot, where the x-axis represented the between-group difference in the change in KCCQ from baseline to follow-up, and the y-axis represented the corresponding treatment effect (expressed as HRs) on the clinical outcome. Each trial was weighted by the inverse variance of the log HR. The coefficient of determination (R^2^) from the weighted regression was calculated to quantify the proportion of variability in treatment effects on the clinical outcome explained by changes in KCCQ. Heterogeneity was assessed using the I^2^ statistic. In the context of meta-regression, I^2^ represents residual heterogeneity, indicating the proportion of between-study variance not explained by the covariate of KCCQ change. We also reported τ^2^ to provide an estimate of the absolute between-study variance. Values of I^2^ < 25% considered low, 25–75% moderate, and >75% high. All analyses were performed using Stata version 19 (StataCorp, College Station, TX, USA), and a two-sided P value of <0.05 was considered statistically significant.

### 2.6 Sensitivity analysis

To evaluate the robustness of our findings, several sensitivity analyses were performed. First, to account for potential overweighting of trials with multiple treatment arms or cohorts (ATMOSPHERE and GALACTIC-HF), we conducted an analysis including only one independent comparison per trial (the aliskiren plus enalapril arm for ATMOSPHERE and the outpatient cohort for GALACTIC-HF). Second, we performed analyses restricted to trials reporting the Clinical Summary Score and stratified by assessment timing (4–8 months and 12 months). Third, we conducted a sensitivity analysis excluding trials judged to have a high risk of bias in KCCQ data completion. Finally, leave-one-out sensitivity analyses were performed to assess the influence of individual trials on the overall results.

### 2.7 Ethical considerations

This meta-analysis was based entirely on published literature. Ethical approval was not required because this study did not involve any direct interaction with human subjects.

## 3 Results

**Fig. 1:**
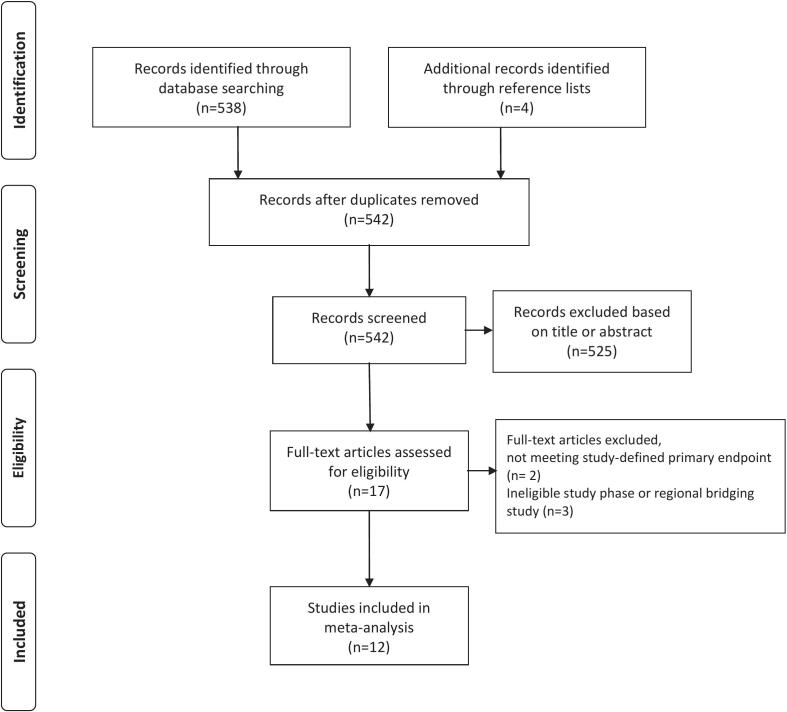
PRISMA flow diagram of study selection.

The study identification and selection process is summarized in Fig. 1. A total of 12 RCTs investigating pharmacological therapies for chronic HF were included in the analysis. The specific reasons for the exclusion of potentially relevant studies are provided in Supplementary Table 1.

The characteristics of the included trials are presented in Table 1. Eight of these trials enrolled patients with HF with reduced EF [13], [14], [15], [16], [17], [18], [19], [20], [21] and four trials focused on patients with HF with preserved EF [22], [23], [24], [25]. Because the ATMOSPHERE trial had two distinct treatment arms and the GALACTIC-HF trial enrolled separate inpatient and outpatient cohorts, both were analyzed as separate datasets in the meta-regression. All included RCTs reported HRs and 95% CIs for the primary composite endpoint and its individual components. Overall, the risk of bias was generally low across most domains (Supplementary Table 2). However, the SHIFT and VICTORIA trials were judged to have a high risk of bias regarding incomplete KCCQ outcome data.

**Table 1:** Study characteristics.

Code	Trial	Year	EF	Active treatment	Control	N	KCCQ score type	KCCQ assessment timepoint (months)	KCCQ available number	Median follow-up (months)
A	SHIFT [13]	2010	≤35%	Ivabradine	Placebo	6505	CSS	12	1681	22.9
B	PARADIGM-HF [14]	2014	≤40%	Sacubitril-valsartan	Enalapril	8399	CSS	8	7706	27
C	TOPCAT [22]	2014	≥45%	Spironolactone	Placebo	3445	OSS	12	2902	39.6
D	ATMOSPHERE [15]	2016	≤35%	Aliskiren	Enalapril	4676	CSS	12	3600	36.6
E	ATMOSPHERE [15]	2016	≤35%	Aliskiren + enalapril	Enalapril	4676	CSS	12	3615	36.6
F	PARAGON-HF [23]	2019	≥45%	Sacubitril/valsartan	Valsartan	4796	CSS	8	4476	35
G	DAPA-HF [16]	2019	≤40%	Dapagliflozin	Placebo	4744	TSS	8	3955	18.2
H	EMPEROR-Reduced [17]	2020	≤40%	Empagliflozin	Placebo	3730	CSS	12	2796	16
I	VICTORIA [18]	2020	≤45%	Vericiguat	Placebo	5050	CSS	4	4401	10.8
J	EMPEROR-Preserved [24]	2021	>40%	Empagliflozin	Placebo	5988	CSS	12	4668	26.2
K	GALACTIC-HF (Outpatients) [19], [20]	2021	≤35%	Omecamtiv mecarbil	Placebo	6148	TSS	6	5419	21.8*
L	GALACTIC-HF (Inpatients) [19], [20]	2021	≤35%	Omecamtiv mecarbil	Placebo	2084	TSS	6	1703	21.8*
M	DELIVER [25]	2022	>40%	Dapagliflozin	Placebo	6263	TSS	8	5795	27.6
N	VICTOR [21]	2025	≤40%	Vericiguat	Placebo	6105	OSS	6	4150	18.5

EF, ejection fraction; KCCQ, Kansas City Cardiomyopathy Questionnaire; TSS, Total Symptom Score; OSS, Overall Summary Score; CSS, Clinical Summary Score.

* Median follow-up for GALACTIC-HF was 21.8 months overall; subgroup-specific values for inpatients and outpatients were not reported.

**Fig. 2:**
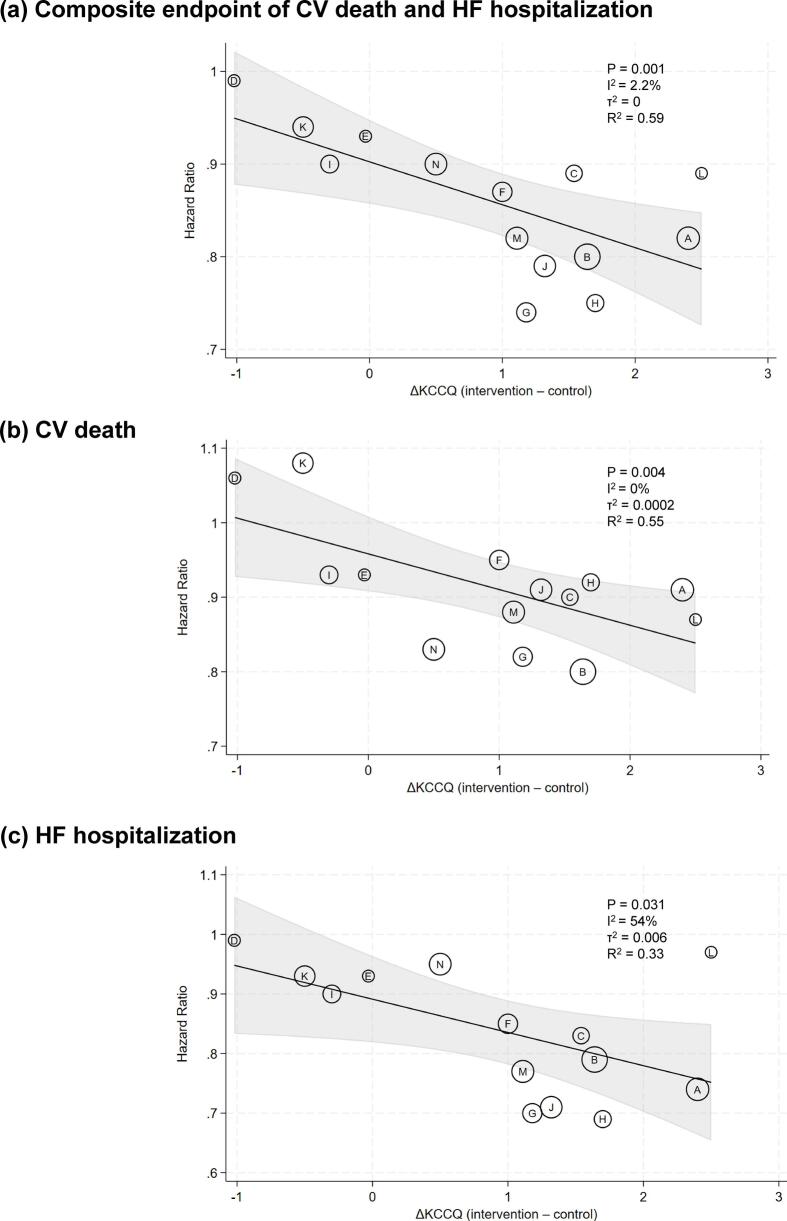
**Association between changes in** t**he Kansas City Cardiomyopathy Questionnaire and clinical outcomes.** Each circle represents a trial, labeled with a letter corresponding to the trials listed in Table 1. The x-axis indicates the between-group difference in the Kansas City Cardiomyopathy Questionnaire (KCCQ) change, and the y-axis indicates the treatment effect on the composite endpoint of cardiovascular (CV) death and heart failure (HF) hospitalization (a), CV death (b), and HF hospitalization (c). Bubble size reflects the trial weight in the random-effects meta-regression. The y-axis is presented on the hazard ratio scale for clinical interpretability, with the underlying regression analysis performed on the log-hazard ratio scale.

Changes in KCCQ scores were significantly correlated with the primary composite endpoint of cardiovascular death and HF hospitalization (regression coefficient [95% CI] = −0.0611 [−0.0930, −0.0292]; p = 0.001; I^2^ = 2.2%; τ^2^ = 0; R^2^ = 0.59; Fig. 2a). Similarly, changes in KCCQ scores were significantly correlated with each component of the composite endpoint when analyzed separately, including cardiovascular death (regression coefficient [95% CI] = −0.0676 [−0.1099, −0.0254]; p = 0.004; I^2^ = 0%; τ^2^ = 0.0002; R^2^ = 0.55; Fig. 2b) and HF hospitalization (−0.0700 [−0.1322, −0.0775]; p = 0.031; I^2^ = 54%; τ^2^ = 0.006; R^2^ = 0.33; Fig. 2c).

Sensitivity analyses confirmed the robustness of the primary findings, with consistent directions of association observed across all models (Supplementary Table 3). The associations remained stable when including only one independent comparison per trial and when restricting the analysis to the Clinical Summary Score. Similarly, consistent directions of association were observed regardless of different assessment timing (4–8 months and 12 months). The findings remained consistent even after excluding the trials judged to have a high risk of bias due to incomplete KCCQ data (SHIFT and VICTORIA). Finally, leave-one-out analyses demonstrated that the overall results were not driven by any single trial (Supplementary Tables 4–6).

## 4 Discussion

We evaluated the association between changes in KCCQ and major clinical outcomes using data from large-scale RCTs that employed a composite of cardiovascular death and HF hospitalization as the primary endpoint. We found that changes in KCCQ were significantly correlated with the primary endpoint and their individual components. These findings highlight the importance of considering KCCQ, a patient-reported outcome measure, as a candidate intermediate endpoint in HF drug development.

In contrast to our findings, Angelico-Goncalves et al. conducted a trial-level meta-regression of RCTs and reported significant correlations between KCCQ changes and treatment effects on the composite endpoint of cardiovascular death and HF hospitalization, as well as on HF hospitalizations, but not on cardiovascular death [26]. We believe that the study by Angelico-Goncalves et al has several limitations. Most importantly, the study included multiple phase 2 trials with relatively short follow-up durations, which likely contributed to the lack of a significant association with cardiovascular mortality. Moreover, since the publication of their report, an additional pivotal phase 3 trial has been published [21]. Our analysis, by contrast, included only phase 3 trials and incorporated this most recent large-scale trial, thereby providing a more robust assessment.

The trial-level coefficient of determination (R^2^ = 0.59) observed for the primary composite endpoint of cardiovascular death and HF hospitalization exceeded the commonly cited threshold of 0.50, which is often referenced in regulatory discussions on surrogate endpoint validation [27]. This finding suggests that changes in KCCQ explained more than half of the variability in treatment effects on the clinical outcome, indicating a moderate to strong association. Although the observed R^2^ reached this benchmark, these results should be considered hypothesis-generating, and KCCQ currently remains a promising candidate intermediate endpoint for supportive evidence. Additional validation, such as individual patient-level data meta-analyses and cross-mechanism validation, will be required to further confirm KCCQ as a formal regulatory surrogate endpoint.

While a 5-point improvement in KCCQ is an established threshold for a minimal clinically important difference representing a patient’s subjective symptom relief [10], it must be distinguished from the magnitude of change required to evaluate KCCQ as a candidate intermediate endpoint for hard clinical outcomes. For instance, a 5-point improvement predicts a substantial reduction in the risk of the primary composite outcome (around 25%; HR 0.75), while a more modest 2-point change is associated with an approximately 10% risk reduction (HR 0.90)—a benchmark often considered a clinically meaningful treatment effect by regulatory authorities [18]. This interpretation assumes linearity and cross-trial comparability.

In addition to patient-reported outcomes such as the KCCQ, physiological measures have also been evaluated as potential surrogate endpoints in HF trials. Among these, BNP or NT-proBNP, markers of ventricular wall stress and filling pressure, has long been used as a putative physiological surrogate endpoint. However, previous trial-level meta-regression analyses showed that changes in BNP or NT-proBNP predicted HF hospitalization but not all-cause mortality [4], [5]. However, these studies were conducted in earlier therapeutic eras and predominantly included trials focusing on HF with reduced EF. Further studies are warranted to evaluate the potential of physiological measures as surrogate endpoints for the composite outcome of cardiovascular death and HF hospitalization, which is commonly adopted in recent phase 3 trials. Furthermore, combining KCCQ with physiological measures may enhance the predictive performance and this integrated surrogacy should be further evaluated using individual patient-level data.

The present study has several important limitations. First, the KCCQ domains and the timing of their assessment were not identical across the included RCTs. However, the domains of the KCCQ are known to be strongly correlated with one another. Additionally, the consistent directions of association observed in our sensitivity analyses restricted to the Clinical Summary Score or for different assessment timepoints (4–8 months and 12 months) suggest that the findings are relatively robust to variations in KCCQ domains and assessment timing. Second, this analysis was conducted at the trial level rather than using individual patient data. Therefore, our findings cannot account for within-trial variability or patient-level heterogeneity, and residual confounding may remain. Future studies using individual patient data meta-analysis are needed to validate and extend our observations. Third, while we included contemporary large-scale trials, the number of available studies remains modest, and ongoing and future trials may further clarify the utility of KCCQ as a candidate intermediate endpoint in HF trials. Finally, our findings are based on pharmacological phase 3 trials using a specific composite endpoint. Thus, the applicability of these results to broader HF development programs, including device-based or non-pharmacological interventions, or to trials using different clinical endpoints, remains to be established.

## 5 Conclusion

In this trial-level meta-regression of recent large-scale RCTs of chronic HF, changes in KCCQ were significantly associated with the primary composite endpoint of cardiovascular death and HF hospitalization, as well as with each component analyzed separately. These findings support KCCQ as a promising candidate intermediate endpoint that provides supportive evidence for the clinical benefit of HF therapies. However, our results remain hypothesis-generating, and further validation using individual patient data and cross-mechanism studies is warranted before KCCQ can be formally established as a regulatory surrogate endpoint.

## CRediT authorship contribution statement

**Hidekatsu Fukuta:** Writing – review & editing, Writing – original draft, Project administration, Methodology, Funding acquisition, Data curation, Conceptualization. **Toshihiko Goto:** Writing – review & editing, Supervision, Data curation.

## Declaration of competing interest

The authors declare that they have no known competing financial interests or personal relationships that could have appeared to influence the work reported in this paper.
