# Supplementary material for: Association between treatment-induced changes in the Kansas City Cardiomyopathy Questionnaire and clinical outcomes in chronic heart failure: a trial-level meta-regression analysis
Source: Int J Cardiol Heart Vasc. 2026 Jan 27;63:101881. doi: 10.1016/j.ijcha.2026.101881 (PMC12865619; doi:10.1016/j.ijcha.2026.101881)
Supplement: Supplementary Data 1 [file mmc1.docx]

**Supplementary Table 1.** Excluded studies and reasons for exclusion.

| Study (author, year, trial name) | Category of exclusion | Specific reason for exclusion |
| --- | --- | --- |
| Anker SD et al., 2009 (FAIR-HF) | Ineligible primary endpoint | Primary endpoints were Patient Global Assessment and NYHA class, not the study-defined composite endpoint. |
| Swedberg K et al., 2013 (RED-HF) | Ineligible primary endpoint | Primary outcome included all-cause death, which did not match our defined composite of CV death and HF hospitalization. |
| Ponikowski P et al., 2015 (CONFIRM-HF) | Ineligible study phase | Phase 2 trial focusing on 6-minute walk test distance. |
| Heidenreich PA et al., 2019 (DEFINE-HF) | Ineligible study phase | Phase 2 trial focusing on NT-proBNP and KCCQ-OS improvement proportion. |
| Tsutsui H et al., 2021 (PARALLEL-HF) | Regional bridging study | A small-scale regional study (N=225) designed to confirm the results of the global PARADIGM-HF trial for Japanese regulatory approval. |
