# Supplementary material for: Association between treatment-induced changes in the Kansas City Cardiomyopathy Questionnaire and clinical outcomes in chronic heart failure: a trial-level meta-regression analysis
Source: Int J Cardiol Heart Vasc. 2026 Jan 27;63:101881. doi: 10.1016/j.ijcha.2026.101881 (PMC12865619; doi:10.1016/j.ijcha.2026.101881)
Supplement: Supplementary Data 2 [file mmc2.docx]

**Supplementary Table 2.** Risk of bias assessment.

| Trial | Random sequence generation | Allocation concealment | Blinding of participants and personnel | Blinding of outcome assessment | Incomplete outcome data (Primary endpoints) | Incomplete outcome data (KCCQ) | KCCQ adjustment status | Selective reporting | Other biases |
| --- | --- | --- | --- | --- | --- | --- | --- | --- | --- |
| SHIFT | Low risk | Low risk | Low risk | Low risk | Low risk | High risk | Adjusted for baseline and covariates | Low risk | Low risk |
| PARADIGM-HF | Low risk | Low risk | Low risk | Low risk | Low risk | Low risk | Adjusted for baseline and covariates | Low risk | Low risk |
| TOPCAT | Low risk | Low risk | Low risk | Low risk | Low risk | Low risk | Adjusted for baseline and covariates | Low risk | Low risk |
| ATMOSPHERE (aliskiren) | Low risk | Low risk | Low risk | Low risk | Low risk | Unclear risk | Adjusted for baseline and covariates | Low risk | Low risk |
| ATMOSPHERE (combination) | Low risk | Low risk | Low risk | Low risk | Low risk | Unclear risk | Adjusted for baseline and covariates | Low risk | Low risk |
| PARAGON-HF | Low risk | Low risk | Low risk | Low risk | Low risk | Low risk | Adjusted for baseline and covariates | Low risk | Low risk |
| DAPA-HF | Low risk | Low risk | Low risk | Low risk | Low risk | Low risk | Adjusted for baseline and covariates | Low risk | Low risk |
| EMPEROR-Reduced | Low risk | Low risk | Low risk | Low risk | Low risk | Unclear risk | Adjusted for baseline and covariates | Low risk | Low risk |
| VICTORIA | Low risk | Low risk | Low risk | Low risk | Low risk | Low risk | Adjusted for baseline and covariates | Low risk | Low risk |
| EMPEROR-Preserved | Low risk | Low risk | Low risk | Low risk | Low risk | Unclear risk | Adjusted for baseline and covariates | Low risk | Low risk |
| GALACTIC-HF  (Outpatients) | Low risk | Low risk | Low risk | Low risk | Low risk | Low risk | Adjusted for baseline and covariates | Low risk | Low risk |
| GALACTIC-HF  (Inpatients) | Low risk | Low risk | Low risk | Low risk | Low risk | Low risk | Adjusted for baseline and covariates | Low risk | Low risk |
| DELIVER | Low risk | Low risk | Low risk | Low risk | Low risk | Low risk | Adjusted for baseline and covariates | Low risk | Low risk |
| VICTOR | Low risk | Low risk | Low risk | Low risk | Low risk | High risk | Adjusted for baseline and covariates | Low risk | Low risk |
