# Supplementary material for: Association between treatment-induced changes in the Kansas City Cardiomyopathy Questionnaire and clinical outcomes in chronic heart failure: a trial-level meta-regression analysis
Source: Int J Cardiol Heart Vasc. 2026 Jan 27;63:101881. doi: 10.1016/j.ijcha.2026.101881 (PMC12865619; doi:10.1016/j.ijcha.2026.101881)
Supplement: Supplementary Data 3 [file mmc3.docx]

**Supplementary Table 3.** Sensitivity analyses.

| Analysis type | Outcome | Number of  datasets (n) | Regression  coefficient | Lower  95% CI | Upper  95% CI | P-value | I^2^ (%) | τ^2^ | R^2^ |
| --- | --- | --- | --- | --- | --- | --- | --- | --- | --- |
| Restricted to CSS only | Composite | 8 | -0.0743 | -0.1183 | -0.0302 | 0.006 | 0 | 0 | 0.87 |
| (excluding OSS/TSS trials) | CV death | 8 | -0.0610 | -0.1179 | -0.0041 | 0.039 | 0 | <0.001 | 0.60 |
|  | HF hospitalization | 8 | -0.1051 | -0.1602 | -0.0501 | 0.003 | 0 | 0 | 0.85 |
| --- | --- | --- |  |  |  |  |  |  |  |
| Independent datasets only | Composite | 12 | -0.0732 | -0.1147 | -0.0317 | 0.003 | 0 | 0 | 0.70 |
| (one comparison per trial) | CV death | 12 | -0.0671 | -0.1287 | -0.0055 | 0.036 | 0 | 0.001 | 0.42 |
|  | HF hospitalization | 12 | -0.1073 | -0.1568 | -0.0579 | 0.001 | 0 | 0 | 0.72 |
| --- | --- | --- |  |  |  |  |  |  |  |
| KCCQ assessment timepoints |  |  |  |  |  |  |  |  |  |
| 4-8 months | Composite | 8 | -0.0455 | -0.1052 | 0.0142 | 0.112 | 29 | 0.001 | 0.37 |
|  | CV death | 8 | -0.0785 | -0.1561 | -0.0009 | 0.048 | 16 | 0.002 | 0.53 |
|  | HF hospitalization | 8 | -0.0265 | -0.1322 | 0.0793 | 0.563 | 62 | 0.008 | 0.06 |
|  |  |  |  |  |  |  |  |  |  |
| 12 months | Composite | 6 | -0.0770 | -0.1360 | -0.0180 | 0.022 | 0 | 0 | 0.83 |
|  | CV death | 6 | -0.0454 | -0.1212 | 0.0304 | 0.171 | 0 | 0 | 0.62 |
|  | HF hospitalization | 6 | -0.1186 | -0.1929 | -0.0442 | 0.011 | 0 | 0 | 0.88 |
| --- | --- | --- |  |  |  |  |  |  |  |
| Excluding trials with high risk | Composite | 12 | -0.0626 | -0.1004 | -0.0249 | 0.004 | 16 | 0 | 0.58 |
| of bias | CV death | 12 | -0.0766 | -0.1219 | -0.0313 | 0.004 | 0 | 0 | 0.67 |
|  | HF hospitalization | 12 | -0.0628 | -0.1325 | 0.0070 | 0.073 | 57 | 0.007 | 0.29 |

CV, cardiovascular; HF, heart failure; KCCQ, Kansas City Cardiomyopathy Questionnaire; TSS, Total Symptom Score; OSS, Overall Summary Score; CSS, Clinical Summary Score.
