# Supplementary material for: Association between treatment-induced changes in the Kansas City Cardiomyopathy Questionnaire and clinical outcomes in chronic heart failure: a trial-level meta-regression analysis
Source: Int J Cardiol Heart Vasc. 2026 Jan 27;63:101881. doi: 10.1016/j.ijcha.2026.101881 (PMC12865619; doi:10.1016/j.ijcha.2026.101881)
Supplement: Supplementary Data 4 [file mmc4.docx]

**Supplementary Table 4.** Leave-one-out sensitivity analysis for the primary composite endpoint.

| Excluded trial | Regression coefficient | Lower 95% CI | Upper 95% CI | P-value | I^2^ (%) | τ^2^ |
| --- | --- | --- | --- | --- | --- | --- |
| SHIFT | -0.0628 | -0.0987 | -0.0269 | 0.003 | 10 | 0 |
| PARADIGM-HF | -0.0584 | -0.0935 | -0.0233 | 0.004 | 8 | 0 |
| TOPCAT | -0.0627 | -0.0955 | -0.0299 | 0.001 | 4 | 0 |
| ATMOSPHERE (aliskiren) | -0.0552 | -0.0936 | -0.0168 | 0.009 | 7 | 0 |
| ATMOSPHERE (combination) | -0.0595 | -0.0935 | -0.0256 | 0.003 | 8 | 0 |
| PARAGON-HF | -0.0613 | -0.0949 | -0.0277 | 0.002 | 10 | 0 |
| DAPA-HF | -0.0582 | -0.0902 | -0.0262 | 0.002 | 0 | 0 |
| EMPEROR-Reduced | -0.0574 | -0.0900 | -0.0248 | 0.003 | 0 | 0 |
| VICTORIA | -0.0640 | -0.0993 | -0.0286 | 0.002 | 8 | 0 |
| EMPEROR-Preserved | -0.0595 | -0.0925 | -0.0264 | 0.002 | 5 | 0 |
| GALACTIC-HF (Outpatients) | -0.0594 | -0.0966 | -0.0223 | 0.005 | 10 | 0 |
| GALACTIC-HF (Inpatients) | -0.0760 | -0.1109 | -0.0412 | 0.001 | 0 | 0 |
| DELIVER | -0.0603 | -0.0938 | -0.0268 | 0.002 | 8 | 0 |
| VICTOR | -0.0607 | -0.0941 | -0.0273 | 0.002 | 9 | 0 |
