# Supplementary material for: Association between treatment-induced changes in the Kansas City Cardiomyopathy Questionnaire and clinical outcomes in chronic heart failure: a trial-level meta-regression analysis
Source: Int J Cardiol Heart Vasc. 2026 Jan 27;63:101881. doi: 10.1016/j.ijcha.2026.101881 (PMC12865619; doi:10.1016/j.ijcha.2026.101881)
Supplement: Supplementary Data 5 [file mmc5.docx]

**Supplementary Table 5.** Leave-one-out sensitivity analysis for cardiovascular death.

| Excluded trial | Regression coefficient | Lower 95% CI | Upper 95% CI | P-value | I^2^ (%) | τ^2^ |
| --- | --- | --- | --- | --- | --- | --- |
| SHIFT | -0.0764 | -0.1211 | -0.0316 | 0.003 | 0 | 0 |
| PARADIGM-HF | -0.0681 | -0.1115 | -0.0246 | 0.005 | 0 | 0.0003 |
| TOPCAT | -0.0678 | -0.1116 | -0.0240 | 0.006 | 0 | 0.0003 |
| ATMOSPHERE (aliskiren) | -0.0589 | -0.1132 | -0.0045 | 0.036 | 0 | 0.0007 |
| ATMOSPHERE (combination) | -0.0697 | -0.1149 | -0.0246 | 0.006 | 0 | 0.0005 |
| PARAGON-HF | -0.0703 | -0.1141 | -0.0265 | 0.005 | 0 | 0.0002 |
| DAPA-HF | -0.0653 | -0.1088 | -0.0219 | 0.007 | 0 | 0.0003 |
| EMPEROR-Reduced | -0.0667 | -0.1109 | -0.0225 | 0.007 | 0 | 0.0005 |
| VICTORIA | -0.0682 | -0.1123 | -0.0242 | 0.006 | 0 | 0.0004 |
| EMPEROR-Preserved | -0.0578 | -0.1024 | -0.0133 | 0.016 | 0 | 0 |
| GALACTIC-HF (Outpatients) | -0.0551 | -0.1007 | -0.0096 | 0.022 | 0 | 0 |
| GALACTIC-HF (Inpatients) | -0.0748 | -0.1212 | -0.0284 | 0.005 | 0 | 0.0001 |
| DELIVER | -0.0691 | -0.1110 | -0.0273 | 0.004 | 0 | 0 |
| VICTOR | -0.0724 | -0.1170 | -0.0277 | 0.004 | 0 | 0.0002 |
