# Supplementary material for: Association between treatment-induced changes in the Kansas City Cardiomyopathy Questionnaire and clinical outcomes in chronic heart failure: a trial-level meta-regression analysis
Source: Int J Cardiol Heart Vasc. 2026 Jan 27;63:101881. doi: 10.1016/j.ijcha.2026.101881 (PMC12865619; doi:10.1016/j.ijcha.2026.101881)
Supplement: Supplementary Data 6 [file mmc6.docx]

**Supplementary Table 6.** Leave-one-out sensitivity analysis for heart failure hospitalization.

| Excluded trial | Regression coefficient | Lower 95% CI | Upper 95% CI | P-value | I^2^ (%) | τ^2^ |
| --- | --- | --- | --- | --- | --- | --- |
| SHIFT | -0.0645 | -0.1325 | 0.0035 | 0.061 | 55 | 0.0066 |
| PARADIGM-HF | -0.0698 | -0.1381 | -0.0014 | 0.046 | 58 | 0.0072 |
| TOPCAT | -0.0708 | -0.1373 | -0.0043 | 0.039 | 57 | 0.0067 |
| ATMOSPHERE (aliskiren) | -0.0623 | -0.1391 | 0.0144 | 0.101 | 57 | 0.0069 |
| ATMOSPHERE (combination) | -0.0667 | -0.1342 | 0.0008 | 0.052 | 57 | 0.0069 |
| PARAGON-HF | -0.0702 | -0.1363 | -0.0042 | 0.039 | 57 | 0.0068 |
| DAPA-HF | -0.0665 | -0.1269 | -0.0061 | 0.034 | 51 | 0.005 |
| EMPEROR-Reduced | -0.0685 | -0.1336 | -0.0034 | 0.041 | 56 | 0.0065 |
| VICTORIA | -0.0708 | -0.1412 | -0.0003 | 0.049 | 57 | 0.0072 |
| EMPEROR-Preserved | -0.0656 | -0.1277 | -0.0035 | 0.040 | 53 | 0.0054 |
| GALACTIC-HF (Outpatients) | -0.1062 | -0.1489 | -0.0635 | <0.001 | 0 | 0 |
| GALACTIC-HF (Inpatients) | -0.0618 | -0.1246 | 0.0009 | 0.053 | 52 | 0.0052 |
| DELIVER | -0.0681 | -0.1408 | 0.0046 | 0.064 | 57 | 0.0073 |
| VICTOR | -0.0677 | -0.1310 | -0.0044 | 0.038 | 54 | 0.0059 |
